# Nox4 Is Dispensable for Exercise Induced Muscle Fibre Switch

**DOI:** 10.1371/journal.pone.0130769

**Published:** 2015-06-17

**Authors:** Juri Vogel, Flávia Figueiredo de Rezende, Susanne Rohrbach, Min Zhang, Katrin Schröder

**Affiliations:** 1 Institute for Cardiovascular Physiology, Goethe-University, Frankfurt, Germany; 2 Cardiovascular Division, King’s College London BHF Centre of Excellence, London, United Kingdom; 3 Physiologisches Institut, Justus-Liebig-Universität, Gießen, Germany; University of Illinois at Chicago, UNITED STATES

## Abstract

**Introduction:**

By producing H_2_O_2_, the NADPH oxidase Nox4 is involved in differentiation of mesenchymal cells. Exercise alters the composition of slow and fast twitch fibres in skeletal. Here we hypothesized that Nox4 contributes to exercise-induced adaptation such as changes in muscle metabolism or muscle fibre specification and studied this in wildtype and Nox4-/- mice.

**Results:**

Exercise, as induced by voluntary running in a running wheel or forced running on a treadmill induced a switch from fast twitch to intermediate fibres. However the induced muscle fibre switch was similar between Nox4-/- and wildtype mice. The same held true for exercise-induced expression of PGC1α or AMPK activation. Both are increased in response to exercise, but with no difference was observed between wildtype and Nox4-/- mice.

**Conclusion:**

Thus, exercise-induced muscle fibre switch is Nox4-independent.

## Introduction

Exercise increases the formation of reactive oxygen species (ROS). Contraction-induced ROS generation has been shown to be an important physiological function for the regulation of both muscle force production and contraction-induced adaptive responses of muscle fibres to exercise training [1]. One important source of ROS in cells is the family of NADPH oxidases, which comprises seven members: Nox1 through 5 and DUOX1 and 2. Among the Nox enzymes Nox4 is an exception. Different to other NADPH oxidases, Nox4 is constitutively active and produces H_2_O_2_ [2]. These features enable Nox4 to elicit long lasting and adaptive signalling processes as involved in differentiation or angiogenesis.

Besides changes in angiogenesis adult skeletal muscle adapts to work load with hypertrophy / atrophy and muscle fibre switch. Depending on their capilarization and metabolic and contractile properties, muscle fibres group into three major categories: Slow twitch type I fibres with high capillary density and high oxidative capacity adapted to endurance exercise, fast twitch fibres type IIb fibres with low capillary density and low oxidative capacity ideal for sprint and anaerobic performance and type IIa fibres, which have an intermediate position. These muscle can work for up to 30 minutes, have intermediate capillary density and high oxidative capacity. The three fibre types differ in their type of myosin which defines the ATPase activity of the muscle. Slow type I fibres express MHCIb and within the two fast types, type IIA expresses MHCIIa, type IID MHCIIx, and type IIb express MHCIIb [3]. Since long, it is debated on whether or not the fiber pattern within one muscle is genetically determined. A landmark study comparing fiber types in monozygotic and dizygotic twins provided strong support for a genetic determination of muscle fiber composition in humans [4]. Nevertheless, conversion of type IIB into type hA fibers with intensive endurance training has been demonstrated [5] and leg immobilization decreases the percentage in type I fibers [6]. Moreover, in addition to the standard fiber type nomenclature, a variety of hybrid fibers can be distinguished, and their phenotypic variation is less well studied as they are not covered by current categorization. Thus, genetic determination as well as demand impact on the fiber composition. Indeed muscle fibres are capable of altering their phenotype in response to changes in demand, e.g., increased or decreased neuromuscular activity [7], mechanical loading or unloading [8], altered hormonal profiles (especially of the thyroid hormones [9]), and aging [10]. Already some training units are sufficient to induce a reduction in type IIb fibres and a corresponding increase in type IIa fibres together with a switch in MHC isoforms [11,12].

Exercise-induced gene expression is at least in part a consequence of an increase in free intracellular Ca^2+^ as a consequence of more frequent neural stimulation. Fibre-type-specific gene expression in skeletal muscles has been described to be controlled by the calcium-regulated serine/threonine phosphatase calcineurin. Activation of calcineurin in skeletal myocytes selectively up-regulates slow-fibre-specific gene promoters, while inhibition of calcineurin promotes slow-to-fast fibre transformation. Transcriptional activation of slow-fibre-specific transcription appears to be mediated by a combinatorial mechanism involving NFAT and MEF2 [13]. In a previous work we found that Nox4 contributes to the increase in intracellular Ca^2+^ in the course of osteoclast differentiation [14]. Others found that in skeletal muscle Nox4-derived H_2_O_2_ directly controls the cytosolic calcium concentration during tetanic contraction providing a potential link between Nox4 and muscle adaptation [15]. On this basis, we hypothesize that Nox4 contributes to the switch of fast to slow muscle fibres in response to exercise.

Utilizing three different regimens of exercise herein we analysed the contribution of Nox4 to muscle fibre switch in wildtype and Nox4-/- mice.

## Material and Methods

### Animals

All animal experiments were conducted in accordance with the German Animal Protection Act and were approved by the District Government of Darmstadt (approval numbers V54-19c20/15-F28/31 and-F28/23) Germany. Animals in this study where killed by cervical dislocation after isofluran (Forene, AbbVie) anaesthesia. C57/BL6 Nox4^-/-^ mice have been previously described [16]. Animals had been backcrossed for 10 generations onto the C57BL6/J background and C57BL/6J mice served as controls. All experiments were initiated at a mouse age of 6–8 weeks and only male animals were used. Mice were housed in a specified pathogen-free facility with 12/12 hours day/night cycle and free access to chow and water. Body weight was monitored at least at the beginning and at the end of the experiments.

### Animal models

Treadmill exercise training was performed on a 4-chamber running belt system (TSE). For repeated forced endurance exercise mice were trained daily for 1h with additional warm-up and cool-down phase. Two different protocols were used: A short, more severe and a longer, more moderate one: The 10 days training was performed initially at 10 m/min and a 5% incline with a gradual increase to 15m/min and 10% incline equal for all mice. The 7 weeks training was performed 5 days/week followed by 2 days break during the weekend. Within the first two weeks treadmill speed was gradually increased from 10 m/min with 5% incline to 15m/min and 10% incline. The rational for having 10 days vs. 7 weeks treadmill was to have an extreme early time point and a time point that for sure will represent a phenotype of regular training induced changes. Mice in the control groups remained in their cages in the treadmill room throughout the exercise bouts. For the voluntary running experiment, mice randomly assigned to the 4 weeks running group (n = 6–8) were provided with a running wheel equipped with an activity counter (running distance). It would be an oversimplification to assume that treadmill running and voluntary running in a running wheel only differ in the intensity of exercise. Numerous other factors are of relevance here: Wheel running is a burst exercise, which occurs throughout the whole night, it is not associated with the psychological stress of the treadmill and happens at the physiological circadian activity maximum of the mice. At the end of the experiments, mice were sacrificed immediately after the last training and muscles were quickly excised, rinsed with ice-cold PBS (phosphate buffered saline), blotted dry, snap-frozen, and stored in liquid nitrogen or TissueTek for later analyses.

### Histochemical analysis of skeletal muscle

To determine the muscle fiber-type composition, myofibrillar adenosine-triphosphatase (mATPase) histochemistry was performed following the method of Brooke and Kaiser [17]. Briefly 10μm thick sections were pre-incubated at pH 4.3 in Na-Acetate/ KCl buffer, 40 mmol/L each. ATPase reaction was allowed at pH 9.4 with ATP (adenosine triphosphate, 1.6g/l) followed by sequential incubation with 1% Ca^2+^, 1% CoCl_2_ and eventually staining with 1% (NH_4_)_2_S. As a result type 1 (slow) fibres appear darkest, type IIb (fast) intermediate, and type IIa lightest.

### Analysis of mRNA expression

Total RNA was extracted from the muscle tissue with TRIzol according to the manufacturer's instructions (Qiagen). From 1 μg of RNA cDNA synthesis was carried out with SuperScript III Reverse Transcriptase (Invitrogen, Carlsbad, CA, USA) and random hexamer primers; semiquantitative real-time PCR was performed with Fast Plus EvaGreen Master Mix for qPCR w/Low ROX (2x, 100 rxn) (Biotium, Hayward, CA, USA) in a Mx3005 cycler (Stratagene) with the indicated primers. We tried several standard housekeeping genes like EF (eukaryontic elongation factor), GAPDH (glycerinaldehyd-3-phosphat-dehydrogenase) or β-actin and all of them where regulated upon exercise. Eventually we found B2M (beta-2-microglobulin) to be stable expressed in all forms of exercise performed by the mice. Relative expressions of target genes were normalized using B2M as housekeeping gene, analysed by the delta-delta-CT method and given as ratio compared to control experiments. The following primers were used:

**Table 1:** 

Target	Sequence 5'-NNN-3'	reference, if applicable
mMyHCI fw	GCCTGGGCTTACCTCTCTATCAC	[18]
mMyHCI rev	CTTCTCAGACTTCCGCAGGAA	
mMyHCIIa fw	CAGCTGCACCTTCTCGTTTG	
mMyHCIIa rev	CCCGAAAACGGCCATCT	
mMyHCIIx fw	GGACCCACGGTCGAAGTTG	
mMyHCIIx rev	CCCGAAAACGGCCATCT	
mMyHCIIb fw	CAATCAGGAACCTTCGGAACAC	
mMyHCIIb rev	GTCCTGGCCTCTGAGAGCAT	
mB2M fw	GTCTTTCTGGTGCTTGTCTC	
mB2M rev	GTATGTTCGGCTTCCCATTC	
mPGC1alpha fw	ACAGCTTTCTGGGTGGATTG	
mPGC1alpha rev	TGTCTCTGTGAGAACCGCTA	
mGLUT4 fw	ATGGCTGTCGCTGGTTTCTC	[19]
mGLUT4 rev	ACCCATGCCGACAATGAAGT	
mCytochrom B fw	CAATCGTTCACCTCCTCTTC	
mCytochrom B rev	TCTGGGTCTCCTAGTATGTC	

### Statistical analysis

Unless otherwise indicated, data are given as means ± standard error of mean (SEM). Statistical analysis for multiple groups was performed by analysis of variance (ANOVA) followed by Bonferroni LSD-post-test and for two group comparisons by two-tailed T-test for normally distributed values. Not normally distributed values were analysed by Mann-Whitney-Test. A probability value < 0.05 was considered significant.

## Results

### Exercise-induced muscle fibre switch is independent of Nox4

**Fig 1:**
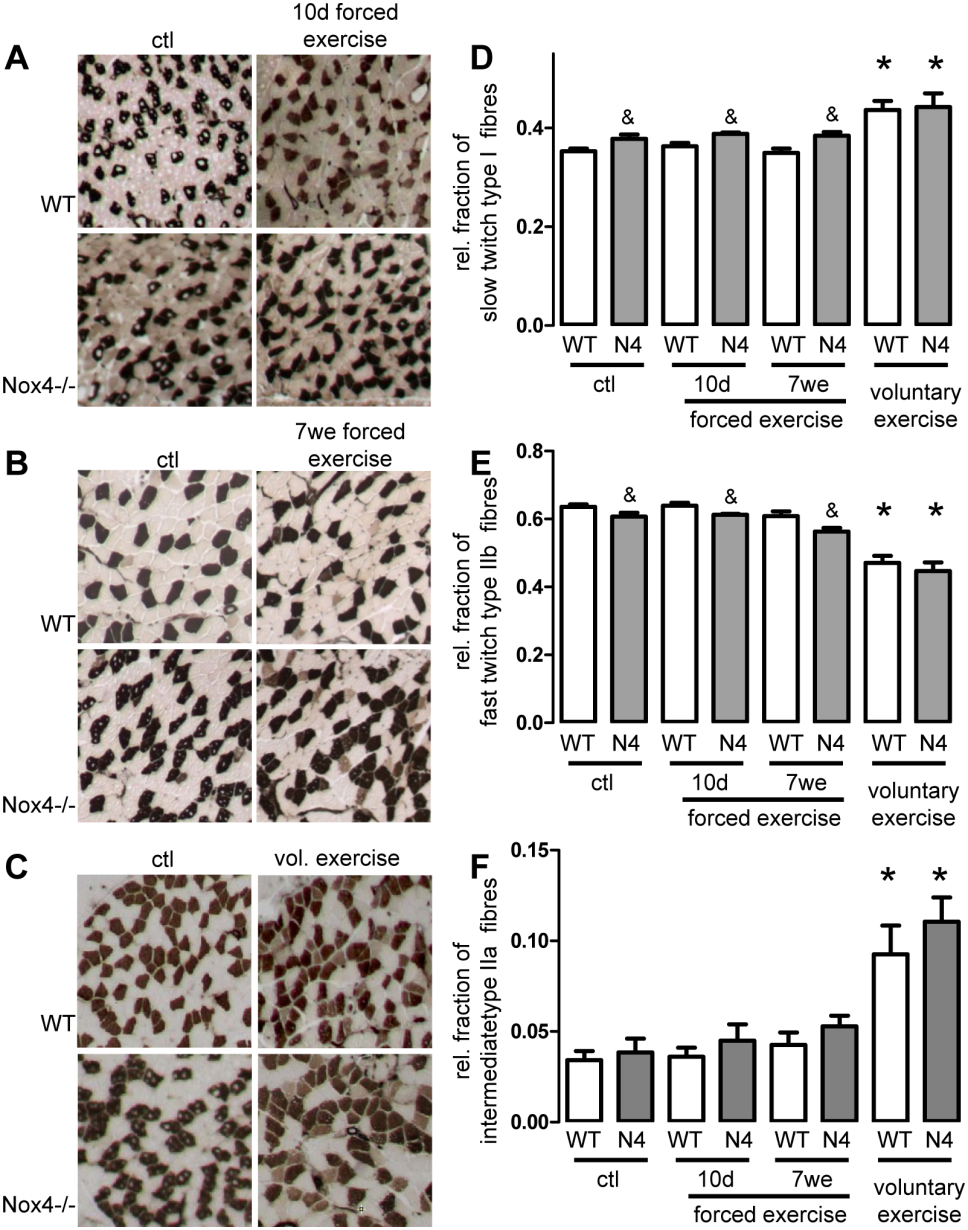
Exercise-induced muscle fibre switch is independent of Nox4. (A-C) Representative images of sedentary and exercised soleus muscle stained for myosin ATPase to determine fibre-type distribution. (D-F): Quantification of slow (dark), fast (light) and intermediate fibres ratio per field of view. mean±SEM (n>5). *p <0.05 (ctl vs. Ex.); &p<0.05 (WT vs. Nox4-/-)

Fibre distribution was analysed by ATPase staining. As shown in Fig 1, the relative number of slow twitch fibres was slightly higher in sedentary Nox4-/- animals when compared to wildtype mice. Although the numeral difference in muscle composition was rather small, it appears that Nox4 deficiency may lead to greater expression of slow fibre type muscle under sedentary conditions (Fig 1). Exercise in mice was performed with three different protocols: 10 days forced exercise, 7 weeks forced exercise and 4 weeks voluntary running. In the voluntary group mice had free access to running wheels and both strains ran similar distances (WT 5646±930m vs. Nox4-/- 4352±955m, n = 6, p = ns). Neither short term nor long term repeated forced exercise had an effect on the distribution of the different muscle fibres (Fig 1A, 1B, 1D, 1E & 1F). In contrast, voluntary exercise increased the relative number of slow twitch and intermediate fibres on the cost of fast twitch fibres in the skeletal muscle of both, wildtype and Nox4-/- mice (Fig 1C–1F). Importantly the portion of fast fibres decreased much more than the fraction of slow fibres increased and thus the number of intermediate fibres increased with exercise to a higher extent than the slow fibres (Fig 1F). This is in line with the concept that fast twitch fibres through the intermediate fibre type trans-differentiate into slow fibres or remain at the stage of intermediate fibre type upon exercise. Importantly, the basal difference in fibre composition between wildtype and Nox4-/- animals disappeared upon voluntary exercise. However, the change in the relative composition of muscle fibres was similar between wildtype and Nox4-/- mice, indicating that exercise-induced changes in muscle fibre composition occur independently of Nox4.

**Fig 2:**
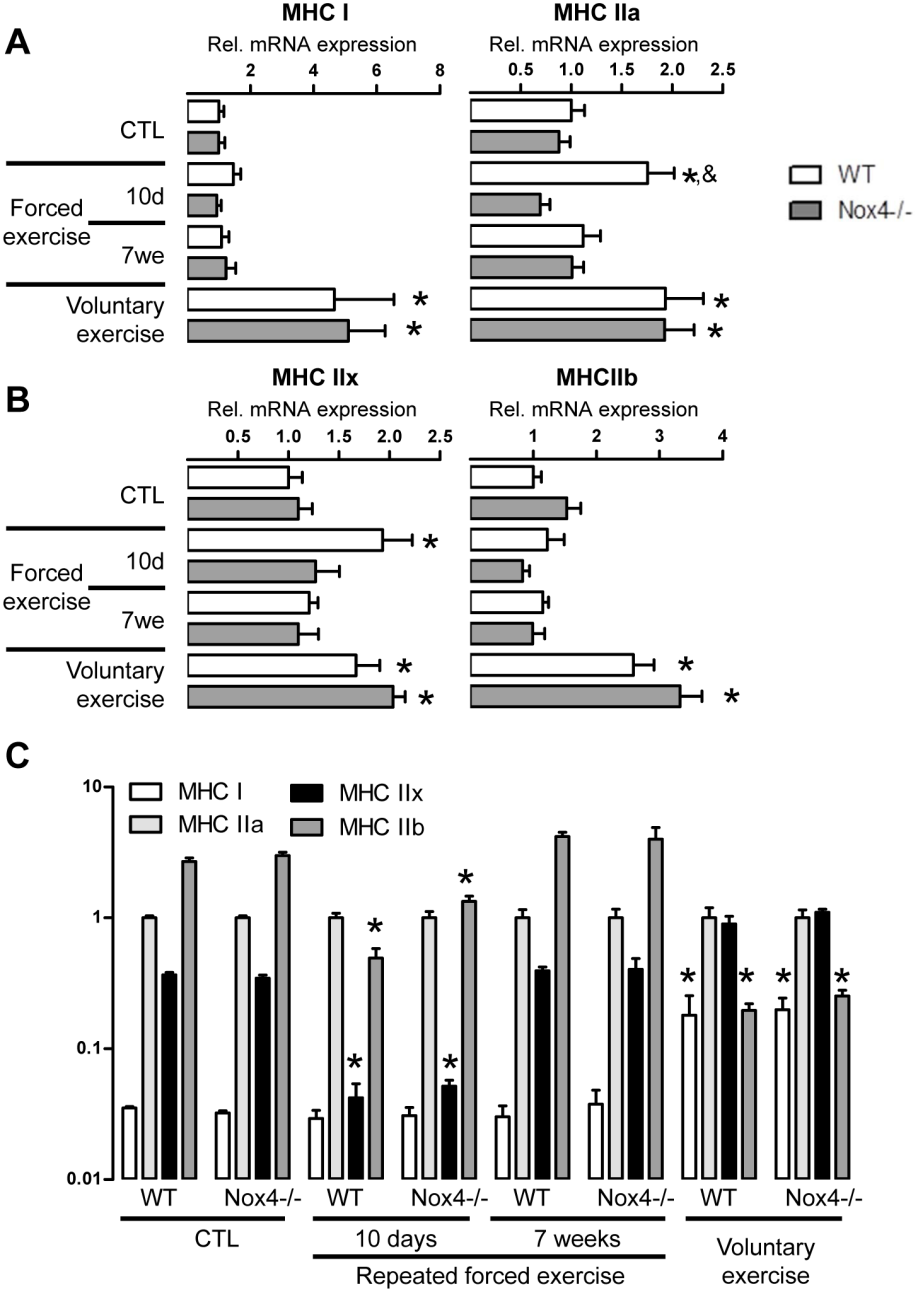
Nox4 has no impact on MHC expression pattern. (A;B) Quantitative PCR for the genes indicated after 10 days and 7 weeks of repeated forced or 4 weeks of voluntary exercise relative to the wildtype controls. (C): Statistics of MHC isoform expression relative to MHCIIa on a logarhythmic scale. mean±SEM (n>5). *p <0.05 (ctl vs. Ex.); &p<0.05 (WT vs. Nox4-/-)

ATPase staining as the only way to determine fibre specification is insufficient. Therefore also skeletal muscle myosin heavy chain (MHC) mRNA isoform expression was determined as the expression of the MHC isoforms serves as marker for muscle fibre specification [3]. All MHC isoforms were up-regulated after voluntary running indicating hypertrophy of the muscle fibres, and the extent of the response was similar between wildtype and Nox4-/- mice (Fig 2A & 2B). Different to histology, mRNA expression analyses revealed, that 10 days treadmill exercise induced a significant increase in MHCIIa and IIx mRNA expression in muscles from wildtype mice, which was not observed in Nox4-deficient animals. For a better visualization of relative changes in MHC isoforms we calculated the expression of the MHC isoforms relative to MHC2a. MHC-ratios were similar between wildtype and Nox4-/- mice (Fig 2C). Collectively, the data suggest that only the voluntary exercise protocol induced sustained changes in muscle fibre composition and that Nox4 does not have an impact on exercise-induced fibre specification.

### Exercise induced switch in energy consumption is independent of Nox4

**Fig 3:**
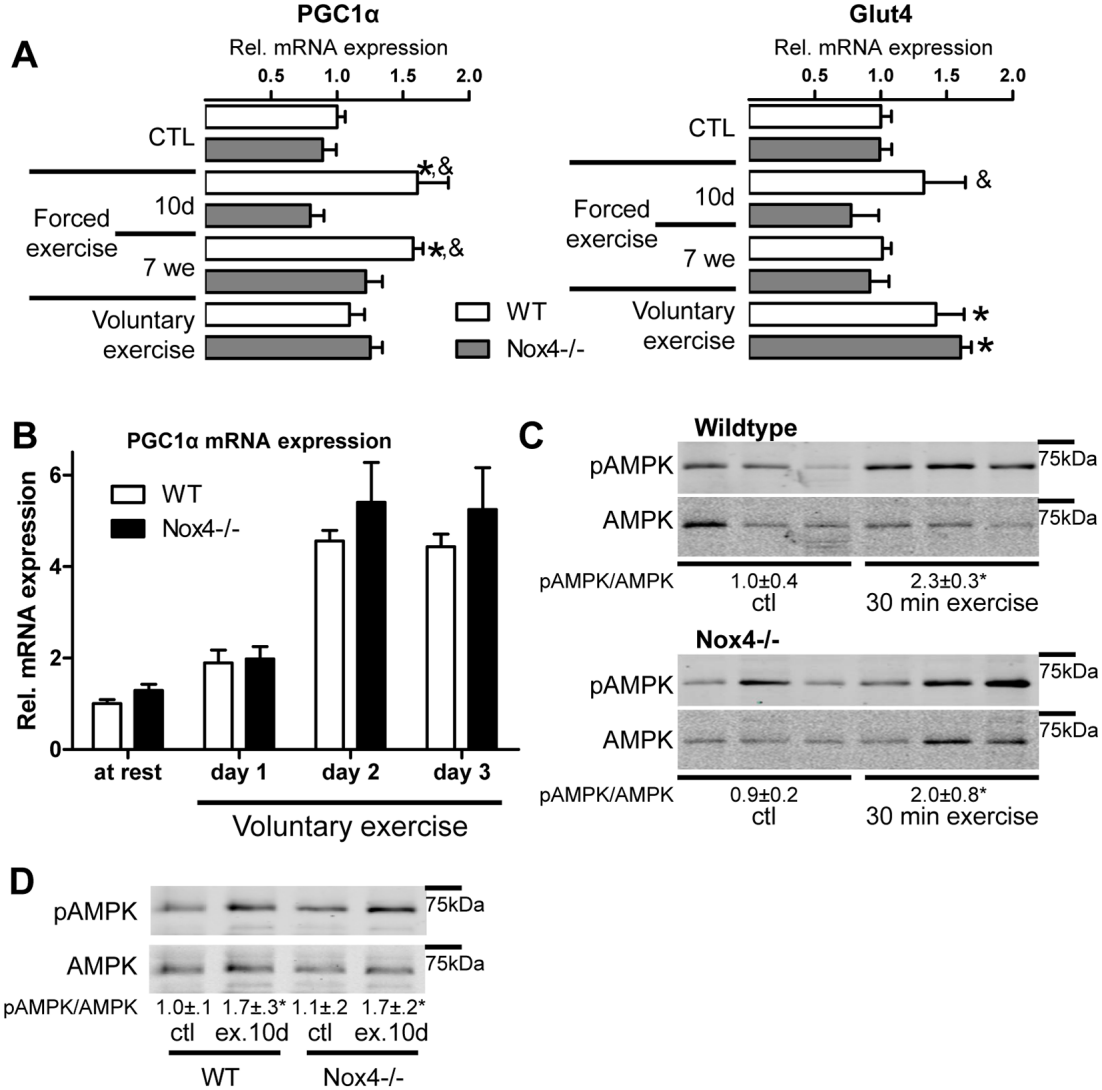
Exercise-induced switch in muscle energy metabolism is Nox4 independent. (A) Quantitative PCR for the genes indicated after 10 days and 7 weeks of repeated forced or 4 weeks of voluntary exercise. (B) Timeline of a quantitative PCR for PGC1α in musculus soleus tissue with voluntary exercise. mean±SEM (n>5). *p <0.05 (ctl vs. exercise); &p<0.05 (WT vs. Nox4-/-) (C&D) Western blot of musculus soleus tissue after (C) 30 of min single or (D) 10 day of repeated forced exercise. Numbers below the blots indicate the ratio of pAMPK and AMPK revealed by densitometry, mean±SEM (n>3). *p <0.05 (ctl vs. Ex.)

Exercise results in metabolic adaptation of the muscle to promote energy supply or-utilization. Energy metabolism and ATP production mainly depend on mitochondria. Interestingly, mitochondrial content as measured by mitochondrial cytochrome B DNA was not different between wildtype and Nox4-/- animals (Fig 3C). However, since one mitochondrion comprises several copies of mitochondrial DNA, we analysed additional markers of energy metabolism and focused on PGC1α. This protein acts as a key mediator of mitochondrial biogenesis in a calcium/ calmodulin-dependent protein kinase IV-dependent manner [20]. As shown in Fig 3A, PGC1α expression was increased upon repeated forced exercise in wildtype but not Nox4-/- mice. This effect was not seen after 4 weeks of voluntary exercise. To further analyse this, we determined PGC1α expression early in voluntary running. PGC1α expression greatly increased after the onset of running but the effect was similar between wildtype and Nox4-/- (Fig 3B). To obtain information about the activity of PGC1α, glucose transporter 4 (GLUT4) mRNA expression was measured, which is under the control of this transcription factor [21]. Repeated forced exercise for 10 days increased GLUT4 expression in wildtype but not in Nox4-/- mice, while the voluntary exercise induced increase in GLUT4 expression was independent of Nox4 (Fig 3A). Next, we analysed AMPK which activates PGC1α. Both 30 min as well as 10 day of repeated exercise increased AMPK phosphorylation independently of Nox4 (Fig 3D & 3E). Thus, Nox4 only has a minor, non-consistent impact on skeletal muscle metabolism control.

## Discussion

Here we provide evidence that Nox4 is dispensable for the exercise-induced muscle fibre switch. Our study exclusively focused on mice and the number of identified fiber types of mice and human is different as well as their distribution and relative contribution to the muscle as large. Thus, caution has to be execute when transferring the current data to the human situation. Nox4 influences muscle fibre composition during development, but this difference is lost after voluntary exercise whereas it is maintained during forced exercise for a short period of 10 days as well as in a long term training over 7 weeks. This also indicates that there is a difference between repeated forced exercise and repeated voluntary running. This is a potential consequence of several factors: The duration, the intensity and the associated stress. Probably, forced exercise reflects submaximal intensity short term load, while voluntary exercise corresponds more to prolonged but moderate training. Although pausing rate on the running belt was identical between the two mouse strains and voluntary running distance was similar, we cannot exclude that the small differences in muscle adaptation are a consequence of minor differences in exercise capacity or intensity. To exclude this point, oxygen update / CO_2_ excretion during exercise should have been measured, what is, unfortunately beyond our capacity. Mitochondria are the central source of energy for muscle contraction, but Nox4 had no influence on mitochondrial density in exercise. Indeed it is known, that at least six weeks of endurance training is required to reach a new, higher steady-state mitochondrial content, dependent on the fiber type being recruited as well as exercise specifications like frequency, intensity and duration [22]. Mice running differs from human running, as mice run is rather an interval running with very fast short distance running followed by a pause. Such kind of running is similar to resistance training, which recruits fast-fibers, does not lead to a mitochondrial adaptation. It rather appears that the very high intensity and low duration of such resistance training represents a strong stimulus for the synthesis of myofibrillar proteins leading to muscle hypertrophy and eventually the mitochondrial content within enlarged muscle fibers may even be “diluted” within the cell [22]. In our experiments there was no difference in the mRNA expression of the key molecule of mitochondrial biogenesis and muscle fibre type determination—PGC1α [23] under basal conditions between the two strains. Upon repeated forced exercise PGC1α, however, was induced in wildtype, but not in Nox4-deficient mice. This effect might be explained by the higher ratio of slow to fast fibers observed under basal conditions in Nox4 deficient mice. Such differences under basal constitution might make fast adaptations in energy metabolism unnecessary as no real deficiency is detected by the sensors. Putative signals coupling muscle activity with gene expression probably arise from combinations of accelerations in ATP turnover or imbalances between mitochondrial ATP synthesis, cellular ATP demand and Ca^2+^ fluxes [22]. In cell culture depletion of intracellular Ca^2+^ stores with ionomycin contributes to the formation of slow fibers and increases mitochondrial activity [24]. Indeed Nox4 regulates ryanodine receptor Ca^2+^ release and thereby maintains intracellular Ca^2+^ level [15]. However, impaired energy sensing could also be a consequence of attenuated AMPK activation as this kinase is one of the most important energy sensors [25]. The AMP activated protein kinase (AMPK) is inhibited allostericly by creatinine phosphate and therefore sensitive to the energy status of the muscle fibre. AMPK induced genes include muscle GLUT-4, hexokinase, uncoupling protein 3, and some of the mitochondrial enzymes of oxidative phosphorylation [26]. However, no differences were found between wildtype and Nox4-/- in the phosphorylation of AMPK or the expression of Glut4. In conclusion, under sedentary conditions Nox4 deficient mice have slightly more slow that fast twitch fibres but no difference in exercise induced muscle fibre switch was obvious between wildtype and Nox4-/- animals.

## Supporting Information

S1 Fig (A;B) Heart and body weight from mice under basal conditions and after 10 days or 7 weeks of repeated forced or 4 weeks of voluntary exercise. (C): Statistics of heart/ body weight. mean±SEM (n>5). *p <0.05 (ctl vs. Ex.); &p<0.05 (WT vs. Nox4-/-)
(TIF)
