# Supplementary figures and images for: Nox4 Is Dispensable for Exercise Induced Muscle Fibre Switch
Source: PLoS One. 2015 Jun 17;10(6):e0130769. doi: 10.1371/journal.pone.0130769 (PMC4471227; doi:10.1371/journal.pone.0130769)

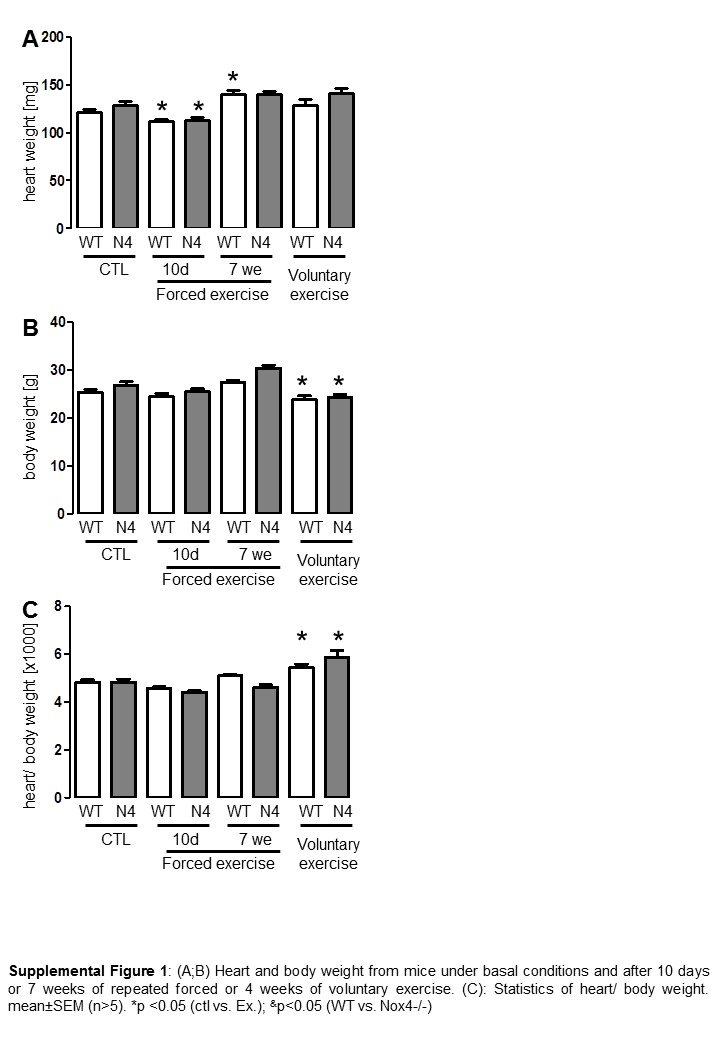

Supplement: S1 Fig — (A;B) Heart and body weight from mice under basal conditions and after 10 days or 7 weeks of repeated forced or 4 weeks of voluntary exercise. (C): Statistics of heart/ body weight. mean±SEM (n>5). *p <0.05 (ctl vs. Ex.); &p<0.05 (WT vs. Nox4-/-) (TIF) [file pone.0130769.s001.tif]
